# eMouseAtlas informatics: embryo atlas and gene expression database

**DOI:** 10.1007/s00335-015-9596-5

**Published:** 2015-08-22

**Authors:** Chris Armit, Lorna Richardson, Bill Hill, Yiya Yang, Richard A. Baldock

**Affiliations:** MRC Human Genetics Unit, IGMM, University of Edinburgh, Edinburgh, Scotland

## Abstract

A significant proportion of developmental biology data is presented in the form of images at morphologically diverse stages of development. The curation of these datasets presents different challenges to that of sequence/text-based data. Towards this end, the eMouseAtlas project created a digital atlas of mouse embryo development as a means of understanding developmental anatomy and exploring the relationship between genes and development in a spatial context. Using the morphological staging system pioneered by Karl Theiler, the project has generated 3D models of post-implantation mouse development and used them as a spatial framework for the delineation of anatomical components and for archiving in situ gene expression data in the EMAGE database. This has allowed us to develop a unique online resource for mouse developmental biology. We describe here the underlying structure of the resource, as well as some of the tools that have been developed to allow users to mine the curated image data. These tools include our IIP3D/X3DOM viewer that allows 3D visualisation of anatomy and/or gene expression in the context of a web browser, and the eHistology resource that extends this functionality to allow visualisation of high-resolution cellular level images of histology sections. Furthermore, we review some of the informatics aspects of eMouseAtlas to provide a deeper insight into the use of the atlas and gene expression database.

## EMAP introduction

eMouseAtlas.org is a free online resource for mouse developmental biology. The eMouseAtlas project (EMAP) created a digital atlas of mouse embryo development as a means of understanding developmental anatomy and exploring the relationship between genes and development. Using the morphological staging system pioneered by Karl Theiler (Theiler 1972), the project has generated 3D models of post-implantation mouse development and used them as a spatial framework for the delineation of anatomical components and for archiving in situ gene expression data in the EMAGE database. There are a number of publications describing the eMouseAtlas resource (Richardson et al. 2014; Armit et al. 2012; Christiansen et al. 2006; Baldock et al. 2003; Davidson et al. 2001). Here we extend these publications and review some of the informatics aspects of eMouseAtlas to provide a deeper insight into the use of the atlas and gene expression database.

EMAP evolved from a demand to be able to capture published, unpublished, and screen-based in situ gene expression data from mRNA hybridisation or immunohistochemistry. To index and analyse the expression patterns, we developed a full spatio-temporal biological atlas, including representative 3D models of the histology of the developing mouse embryo, a basic ontology describing the developing tissues visible in the histology, and delineation of the tissues within the models (3D anatomy domains). This provided a *framework* (Davidson and Baldock 2001) enabling a database of the spatial expression patterns, not constrained by the current understanding of anatomy, and being able to capture any pattern including expression strengths and gradients.

The eMouseAtlas interface also provides essential information for researchers that are new to the field of mouse embryology, including details on how to stage mouse embryos using morphological criteria such as the formation of limb, neural tube, and craniofacial components. In collaboration with the MGI gene expression database (GXD), the EMAP further developed the EMAP anatomy ontology (Hayamizu et al. 2013). This detailed ontology describes the class (*is*-*a*) and partonomic (*part*-*of*) anatomical relationships that exist in the developing mouse embryo and is recognised as the standard ontology in this field. It is under continuous development to include extended detail and lineage information.

**Fig. 1:**
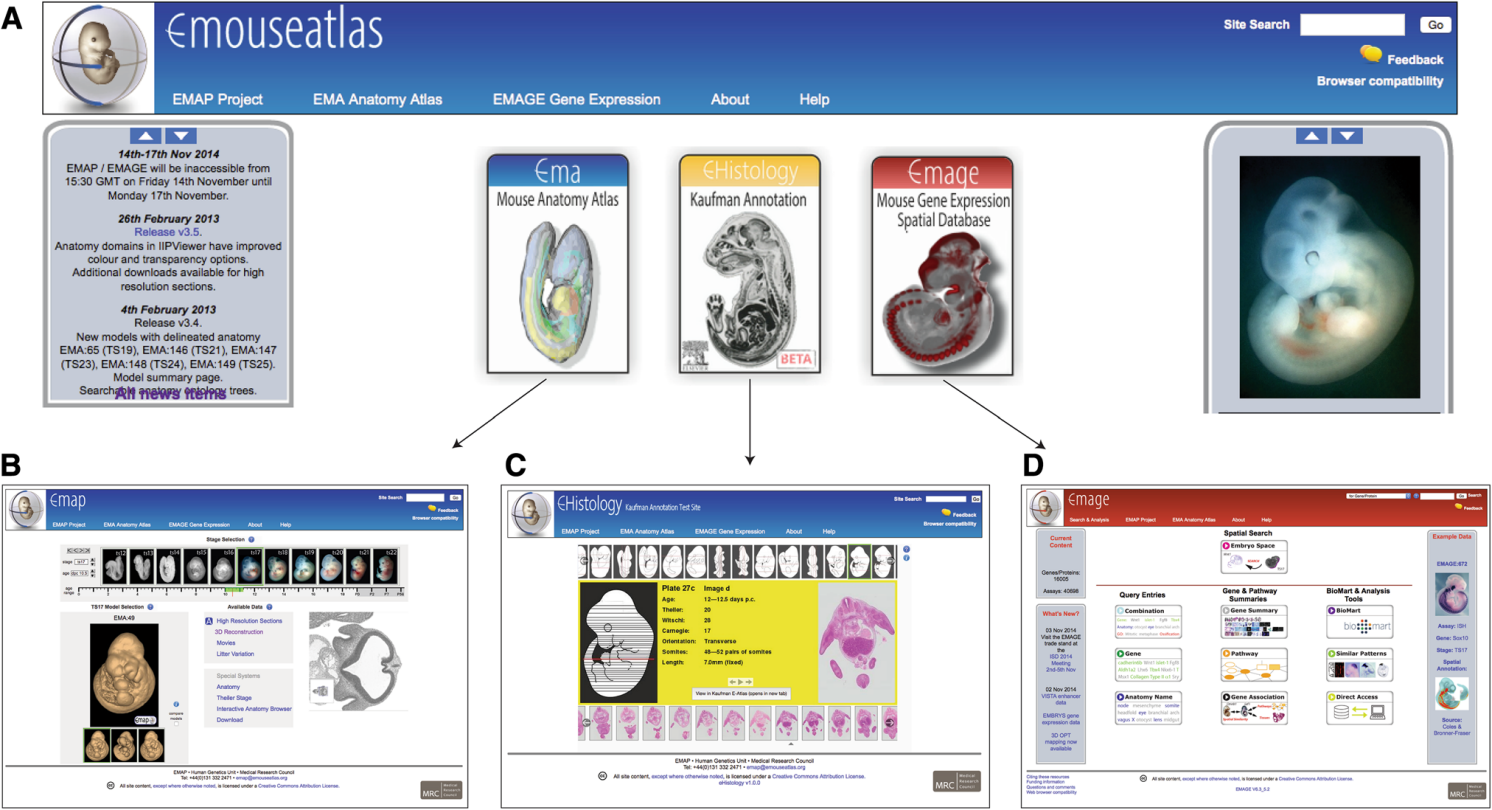
The three main components of eMouseAtlas can be accessed from the eMouseAtlas portal page (**a**). These are EMA (mouse anatomy atlas) (**b**), eHistology (online access to histology images and annotations) (**c**), and EMAGE (gene expression database) (**d**)

The eMouseAtlas resource web portal page (www.emouseatlas.org) provides three primary routes to the data: the mouse anatomy atlas (EMA), the online histology with Kaufman annotations (eHistology), and the gene expression database (EMAGE), which are selected from the portal page (see Fig. 1).

## Mouse anatomy atlas (EMA)

Figure 1b shows the anatomy atlas front page, as accessed from the eMouseAtlas resource web portal page. For each Theiler stage, there are a number of 3D reconstructions and optical projection tomography (OPT) images that can be found using the “stage selector” at the top of the page. For a given stage, the models available are shown in the model selection feature as a browsable “film-strip.” Once selected, the data available for each model are displayed as a series of links. These data include the original high-resolution sections of a 3D reconstruction, delineated anatomy if available, information on littermates, and movies. For each stage, there are also links to morphology-based Theiler stage descriptions, a detailed anatomy ontology and, if available at a particular stage, 3D reconstructions of organ systems within the embryo. These are termed “special systems” and include forelimb, hindlimb, and kidney. In addition, each stage is linked to a stage-matched eHistology viewer with Kaufman Atlas annotations (see below). Furthermore, a download page lists the available 3D reconstructions and 3D anatomy domains that can be downloaded for application-based visualisation.

**Fig. 2:**
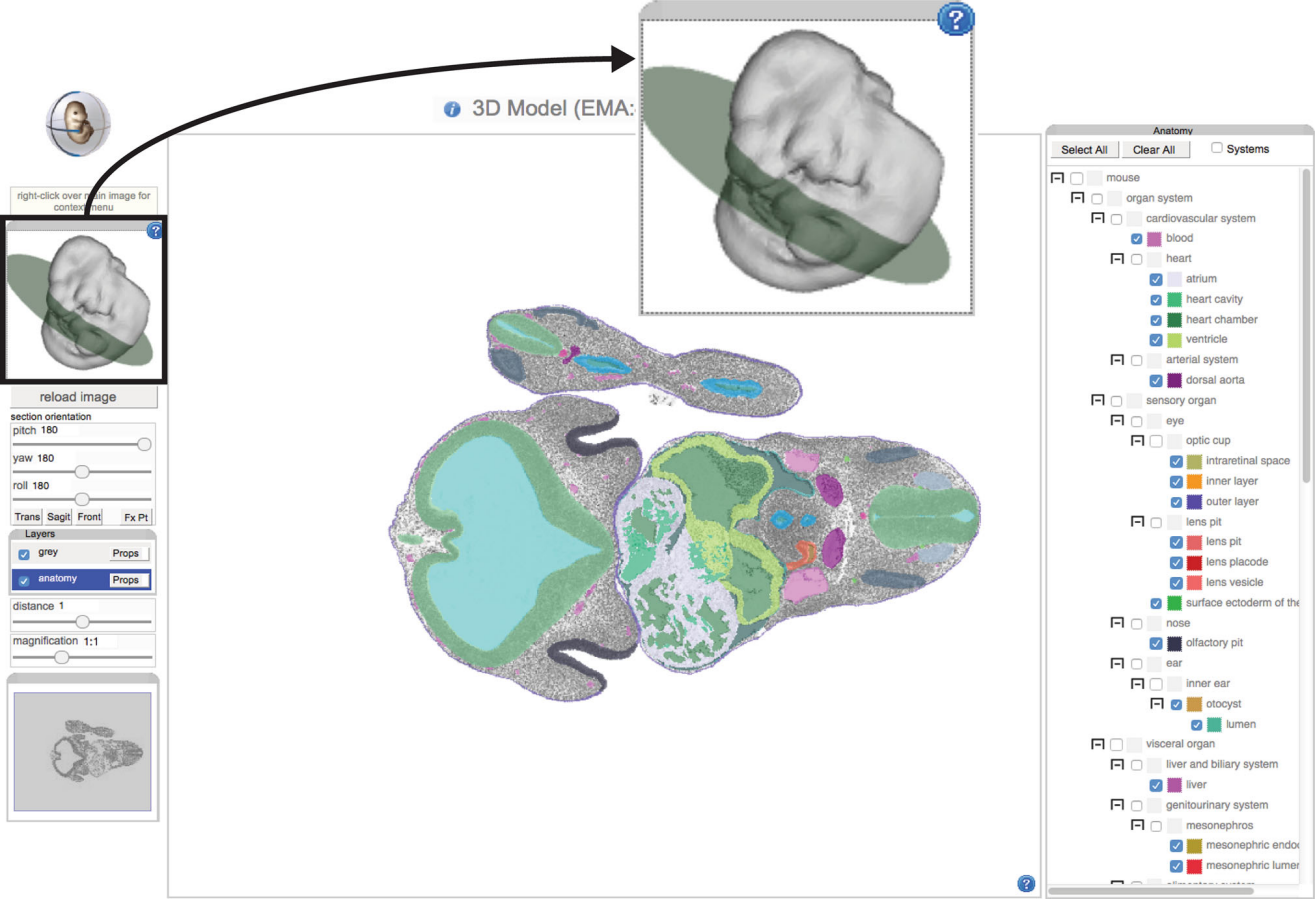
This 3D reconstruction of an eMouseAtlas TS17 embryo is visualised using an IIP3D web tool. The X3DOM interactive 3D surface display (*inset*) allows the user to easily navigate through the 3D space of the models with the green disc delivering feedback on the plane of section. The IIP3D viewer (*centre panel*) allows users to interactively view anatomy domains. Manipulations that can be performed on the displayed image include pan-and-zoom; translating the viewing plane (i.e., distance); and rotating the viewing plane in three dimensions (pitch, yaw, roll). There is the additional option to select layers (*right panel*), and this allows visualisation of delineated anatomical domains

A recent innovation on the EMAP site is the use of an IIP3D viewer (Husz et al. 2012) to allow a user to interactively explore 3D anatomy in the context of a web browser (Fig. 2). This viewer is coupled with a X3DOM navigation tool to allow a user to choose an arbitrary section through a 3D reconstruction and visualise multiple anatomical domains on a single section. For most users, we anticipate that this feature will negate the need to download embryo models and anatomy domains.

## Annotated histology (eHistology)

**Fig. 3:**
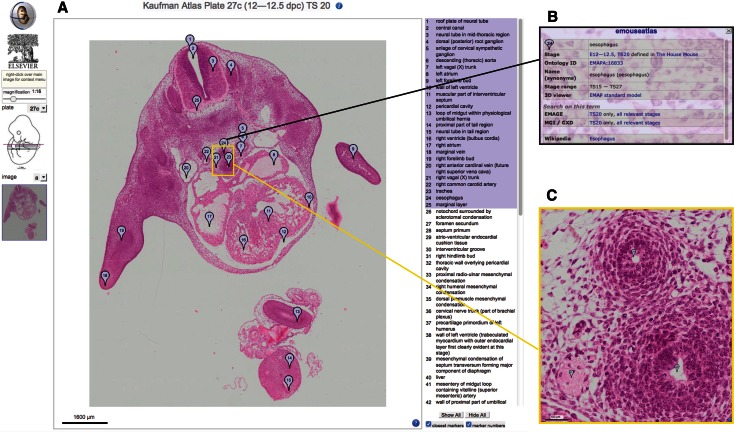
The eHistology interface (**a**) consists of a main panel showing the chosen section with the list of annotations relating to the selected plate and image shown on the *right hand side*. The user is able to choose to only see closest marker pins to the cursor, or to “show all” pins on the current view. Left clicking on any marker pin will open a pop-up window (**b**) containing further information and links relating to the structure defined by that marker pin. There are navigation and magnification controls on the *left hand side* of the main panel. These allow the user to navigate between sections as well as to zoom in on any region of the section to cellular resolution (**c**)

The original concept for the EMAP project stemmed from the publication in 1992 of Matthew H. Kaufman's “The Atlas of Mouse Development” (Kaufman 1992). The original atlas reconstructions used the section series that were generated for the book, and the draft ontology was initiated from the set of annotation terms. Very recently the set of histology sections photographed for the original atlas have been re-digitised at *cellular* resolution and in colour to form the eHistology resource (Graham et al. 2015) (Fig. 3). This resource provides free access to all of the sections of the original Atlas with annotations from the book made visible as map pins in the zoomable images. This allows visualisation of section data from *whole*-*embryo* resolution to *cellular* resolution. Figure 3 shows this interface. These images with the original plate numbering and annotations are provided freely by agreement with Elsevier. The work to deliver this resource involved identifying ontology terms and extending the ontology as needed for each annotation term making it possible to use the eHistology views to query EMAGE and MGI/GXD in terms of anatomy and gene expression. In addition, each of about 10,000 annotations had to be captured and stored in a database; each location marked by Kaufman was captured and re-annotated in the corresponding new image. For users who are unfamiliar with embryo anatomy, we further provide links to Wikipedia entries for anatomical terms. In future versions, we plan to develop an interface that allows for community annotation, thus extending the detail available using expertise from outwith the eMouseAtlas team.

## Gene expression database (EMAGE)

To spatially index expression data, EMAP developed the gene expression database EMAGE, to host raw image data and gene expression pattern annotations. EMAGE archives expression data from in situ hybridization (ISH), immunohistochemistry (IHC), transgene reporter, and enhancer-trap assays of the mouse embryo. These data come from a variety of sources including the published literature, collaborations with large-scale screen projects, and individual submissions from research groups. Many research projects have a condition of funding that data are made publicly available at the end of the project, and the EMAGE database provides a structured and managed mechanism for researchers to satisfy this requirement. In addition, the EMAGE Editorial Office delivers a valuable service by spatially mapping high-impact gene and enhancer expression data, thus allowing these data to be spatially registered, or *mapped*, to the underlying eMouseAtlas embryo models.

The mapping process involves accurate stage selection of mouse embryo models and *spatial warping* of raw image data onto a stage-matched model (Hill and Baldock 2015). This is a means of generating standardised representations of gene expression patterns that can subsequently be archived in the EMAGE database. The mapping process utilises points of morphological equivalence that are defined on a mesh overlaying the data image and the stage-matched model. These points form the basis of a spatial warp that maps the original data image into/onto the space of the reference model. Having defined the warp, the signal in the original image is extracted at various levels of expression by means of colour thresholding. Thus, a representation of the original pattern is defined within the space of the reference model, including information on levels of expression, and without the restriction of anatomical boundaries. Mapping gene expression patterns in this way provides an objective and accurate representation of the expression domains that may extend across multiple anatomical components or be restricted to sub-regions of any one component. In addition, we are able to capture some of the gradient aspect of the pattern and can correct for common artefacts such as trapping. Describing such expression patterns using only text-based descriptions is an extremely laborious (and sometimes impossible) process and requires the use of multiple qualifying statements to accurately describe a complex expression pattern. An additional advantage of this spatial mapping process is that expression patterns can be analysed *spatially* in relation to the expression of other genes, allowing gene expression patterns to be ranked and clustered by spatial similarity or any other spatial relationship. Towards this end, EMAGE has developed web interface tools that allow users to perform queries across the spatially mapped datasets.

## Embryo space query tool

**Fig. 4:**
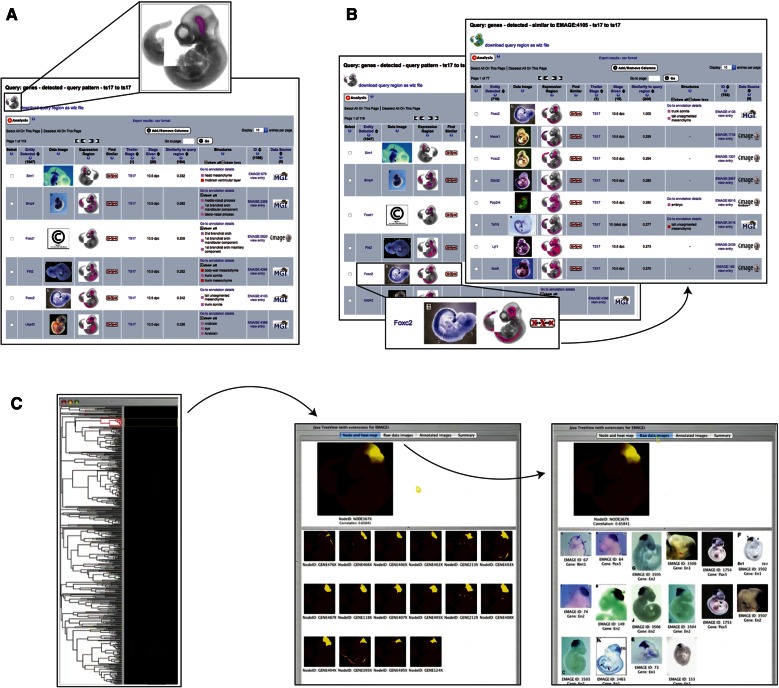
EMAGE “embryo space” query tool (**a**) allows users to “paint” a query region on a model (*inset*). This painted region is used to query the database for mapped patterns that overlap with the query region. These results are ordered by similarity to the query region. The “find similar” tool (**b**) takes a chosen entry (*inset*) and uses the mapped pattern for that entry to query for syn-expression in other entries. The results are ranked by similarity to the query domain, in this case the mapped pattern for the chosen gene (Foxc2). The “spatial clustering” tool (**c**) allows users to select a node in a tree view of spatially similar patterns (*left*). This node can be viewed as a spatial heatmap of expression (*middle*). There is the additional option to view this heatmap alongside the composite of original images that were used to generate a particular node

The principal tool in this respect is the “Embryo Space” application (Fig. 4), whereby a draw tool is used to delineate a region of interest on the embryo model, and this region is then used to query the EMAGE database. The spatial search mechanism is based on the local spatial similarity search tool (LOSSST) algorithm which utilises a calculation based on the intersection of the query domain defined by the user and the domain of “detected” expression annotated in the model (Richardson et al. 2010). The Jaccard index or coefficient, defined in set theory, is used to rank spatial similarity to the region of interest by displaying in descending order gene expression patterns with the highest Jaccard scores. The whole mount option uses a lateral projection view of the embryo model as the reference object and users are invited to paint a region of interest directly on this view of the embryo model. A future release will include a section option that uses a browser-based IIP3D viewer to deliver a user-defined section through the 3D model. Users can then paint a region of interest on this section, and there will be the additional option to paint on multiple sections and thus generate a 3D region of interest. The Embryo space query tool provides a quick and flexible method of querying the EMAGE database in a spatial manner. It is particularly useful for retrieving gene expression patterns that have not been described using text-based (ontology) descriptions.

## Find similar spatial query

The “Find Similar” query uses the LOSSST index to score spatial similarity between mapped gene expression patterns (Fig. 4). The salient difference between this and the “Embryo Space” query tool is that this option does not allow a user to define an arbitrary region of interest but rather uses the mapped gene expression pattern itself as the query domain. Consequently, the “Find Similar” tool will return a list of mapped gene expression patterns ranked by similarity to the gene expression pattern that was queried. At the top of this list, it will return itself as this has the highest possible Jaccard coefficient (intersection/union) of 1. Following this, there will be the most similar gene expression patterns based on a measure of global similarity. It is important to recognise that the “Find Similar” query was designed to capture gene expression patterns that are *co*-*expressed* in regions of the developing mouse embryo and thus occupy the same coordinates in the spatial map. As a means of finding patterns that are globally similar but not necessarily mapped to an identical set of coordinates in the embryo models, we developed the concept of spatial clustering.

## Spatial clustering

Spatial clustering allows users to detect syn-expression groups. As with the “Embryo Space” and “Find Similar” options, this tool does not require any knowledge of specific gene functions, GO enrichments, or gene pathway activities as it is entirely based on the spatial representation of the expression domains. In this way, it is an important discovery tool as it allows genes of unknown function to be clustered alongside very well-characterised genes. Each expression pattern in the set is compared with all other expression patterns in a pair-wise fashion, resulting in a *signature* of Jaccard index similarity scores for each expression pattern. Hierarchical clustering is applied to these signatures, and the clusters are presented as a tree in the EMAGE web interface with each node in the tree represented as a spatial “heatmap” image to highlight the region of syn-expression. The EMAGE web interface allows users to toggle between the heatmap display and views of the original images, and we envisage both options being useful to the end user, with the heatmap display delivering a visualisation of the clustered domain calculation that can be easily compared to the raw image data. Recently, we have applied the same concept of hierarchical clustering to 3D gene expression patterns. In this scenario, 3D gene expression patterns that were captured using the imaging method OPT were spatially mapped onto stage-matched models, and these patterns were hierarchically clustered using exactly the same approach of pair-wise pattern similarities used to generate a syn-expression signature. Spatial clustering is extremely useful for comparing global patterns of gene expression across the entire embryo as it does not require exact co-expression of gene expression patterns in the same voxel space, but rather can find expression profiles that are spatially similar because they share a similar overlap profile. In this context, we envisage this tool as primarily of interest to users wishing to identify spatial relationships between two or more candidate genes and to identify additional genes that may be influencing their expression patterns.

## Image informatics

### Woolz image processing

Woolz image processing software was developed by the MRC Clinical Population and Cytogenetics Unit, now the MRC Human Genetics Unit, for fast microscope slide scanning, chromosome image analysis, pattern recognition, and a wide range of image processing and analysis problems (Piper and Rutovitz 1985). The underlying structures are based on an *interval coding* scheme for binary image domains and the corresponding grey values, and these allow for *fast interval processing*. The Woolz image processing system has been adopted as the standard for the eMouseAtlas Databases and is used for all the reconstructions and anatomical, gene expression, and spatial domains. This is because the interval coding provides significant computing advantages in a range of image processing functions specifically set operations such as *union* and *intersect*, morphological operations such as *erosion* and *dilation*, and other binary image processing such as *distance transforms*, *segmentation,* and *labelling*.

The benefit arises in two ways. The first is that interval coding for a typical anatomy domain or gene expression pattern is more compact than the equivalent binary image representation. The second is that binary operations executed over a domain represented as zero or one within a rectangular region require a comparison for each pixel/voxel location and scale as image area or volume. With interval coding, the comparison is per interval, which scales (on average) with one fewer dimension, i.e., linearly or as the square of the image size, for 2D and 3D images, respectively.

The woolz structures are also compact and, in terms of grey-level data, minimise memory usage *without* compression. From the perspective of the EMAGE database, the major advantage of using Woolz is that it allows for fast calculations of *intersect*, *union, and morphological dilation*, and this allows the Jaccard index (*intersection/union*) and LOSSST value (*intersect, union, dilation, difference*) to be calculated very quickly across large datasets of over 15,000 spatially mapped expression patterns at a single stage of development. Woolz can additionally be used for visualisation of delineated anatomical components and mapped gene expression patterns, and the utility of these is described in the following section.

### Online visualisation of anatomy and gene expression domains

IIP3D is a protocol for delivering image tiles corresponding to a virtual section cut at any orientation through a volumetric image (Husz et al. 2012). The implementation includes a server, configured as a fast-cgi module, and an Ajax/JavaScript viewer to provide a novel fast method to access large 3D volumetric datasets. The mechanism of browsing is similar to Google maps, but instead of a 2D map, 3D volumetric data are displayed. The IIP3D viewer allows any number of layers enabling the co-visualisation of multiple modalities and in particular an anatomy or expression pattern layer. Typical volumetric anatomy viewers use the notion of an “index” image, which encodes the anatomical term as an image value in the 3D volume. This is the mechanism used by Amira (www.fei.com/software/amira-3d-for-life-sciences/) and embedded in the Neuroimaging Informatics Technology Initiative image standard, *NIfTI* (http://nifti.nimh.nih.gov/). This imposes the constraint that no two patterns can share the same locations and is therefore not useful for overlapping patterns such as gene expression or indeed almost any spatially organised data. We therefore use the Woolz *compound object* to include an arbitrary number of overlapping domains (image regions) to be displayed as a combined colour overlay over the grey-level histology image. Any of the domains can be selected for display with individual colours and transparency. The efficiency of the binary processing in Woolz makes it possible to provide a fast and responsive regional overlay interface online for image volumes too large for any typical workstation.

**Fig. 5:**
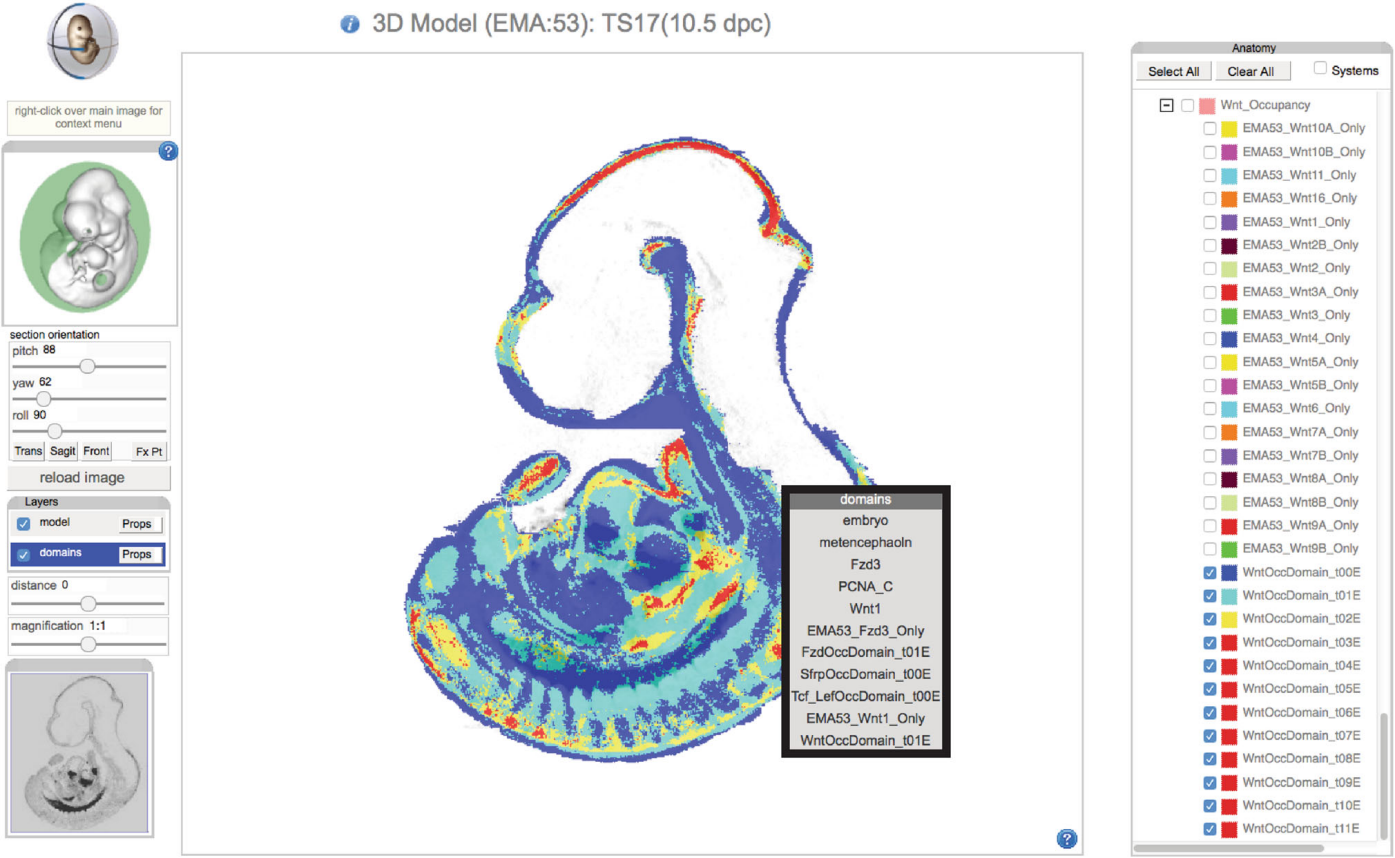
Colour map showing occupancy of spatially mapped Wnt gene expression patterns. An interactive IIP3D viewer is used to deliver a mid-sagittal section through a TS17 model. Spatially mapped Wnt gene expression patterns are visualised as occupancy maps with the number of Wnt patterns per voxel displayed as distinct colours. In this visualisation, the domain of zero occupancy (i.e., no Wnt gene expression) is shown in *blue*, whereas single occupancy (only one Wnt gene) is shown in *green*, dual occupancy (two Wnt genes) is shown in *yellow*, and occupancy scores of three or more patterns (three or more Wnt genes) are shown in *red*. This reveals region of high co-expression (potential signalling centres) and low co-expression within a given gene family. Users are able to interactively explore the occupancy map in more detail using mouseover, which delivers a list of mapped gene expression domains and anatomy domains per voxel

An index defines a region of the 3D image space and the intersection of that 3D region with the current section will return tiles with those pixels. There are additional options to change colour (RGB) and transparency (alpha) for each of the domains. The IIP3D viewer was primarily used by eMouseAtlas as an interactive web interface for visualising 3D anatomy. In this scenario, delineated anatomical components on the various atlas models can be toggled on/off and the colour and transparency values for each layer selected independently and thus modified by the end user to enable bespoke visualisation of regions of interest in the developing mouse embryo. More recently, we have enabled IIP3D as a means of visualising 3D-mapped OPT gene expression patterns. In this case, visualisation is more problematic as various genes may map to a single volumetric pixel, and this is more difficult to visualise using our standard IIP3D display. As a means of overcoming limitations to the number of gene expression domains that can be visualised, we are using *occupancy* per volumetric pixel as a means of displaying the number of gene expression patterns that map to 3D embryo models (Fig. 5). These can be used to filter the data and to allow visualisation of regions of high and low co-expression in a region of interest. An additional development designed for analysis of mapped gene expression domains, now supported, is the ability to retrieve a list of genes that map to each volumetric pixel in a 3D embryo model. The user is provided with an interactive list of tissue component and gene expression as the cursor is passed over the image.

### Applications and data

In building the eMouseAtlas resource, the group has used a series of existing tools and developed, as needed, applications to handle and process the data (see Table 1). The core image processing is undertaken by the Woolz image processing library and associated applications. In addition, we have developed tools for handling and “painting” 3D volumetric images and undertaking complex spatial mapping in both 2D and 3D using the constrained distance transform (Hill and Baldock 2015). Finally, we have developed Java and Python libraries that can access the Woolz image format directly enabling the core data to be used and analysed within tools such as ImageJ. All the code developed under this programme is open source and available from the ma-tech GitHub repository (github.com/ma-tech) with additional information available from www.emouseatlas.org/emap/analysis_tools_resources/software.html.

**Table 1:** Software tools and applications

Application	Description	System
WlzViewer	A viewer application for woolz image objects including 3D grey-level volumes, domains, and surfaces. Multiple objects can be read in and compared	Linux
MAPaint	The primary application for delineating domains and anatomical regions in 3D image data. MAPaint is also the tool for mapping 2D gene expression data by section selection, warping, and colour-segmentation	Linux OSX
MA3DView	A companion application to MAPaint for reviewing 3D image data enabling cropping and interactively controlled grey-level re-mapping	Linux OSX
WlzWarp	A tool for mapping 3D image volumes using the constrained distance transform. This was developed for mapping 3D gene expression data to the EMAP models and can be used for any data. Tools include generation of 3D meshes for enabling the transforms and management of multiple volume views and overlays	Linux
JAtlasViewer	A Java viewer for EMAP models providing a 3D rendering of selected anatomical components with section-based views similar to MAPaint	Linux OSX Windows
Woolz	A C-coded image processing library with a set of command-line executables. The executables enable pipeline processing of image data, bespoke calculations, and image format conversion. The library provide all of the functionality needed within the database and applications	Linux OSX Windows
JavaWoolz	Java woolz—JNI library binding the C-coded woolz dynamic library	Linux OSX Windows
RWoolz	R-bindings for the woolz library	Linux
PyWoolz	Python bindings for the woolz library	Linux
WlzIIPSrv	IIP3D fast-cgi (fcgi) server for delivering tiles through 3D data volume for web browser-based display	Linux OSX

All data made available via the web interfaces are held in the Woolz image format to ensure maximum efficiency and no loss of spatial registration (see Table 2). Data can be downloaded in this format and converted to a range of standard formats such as the open standard NIfTI (http://nifti.nimh.nih.gov/), jpeg and tiff. We will also provide the data pre-converted to NIfTI for convenience and to capture the anatomy delineations as an indexed volume with associated labels and IDs. The following tables summarise the applications, tools, and data available for download.

**Table 2:** Image data available for download

Data	Description	Format
EMAP atlas model	3D reconstruction of each stage from serial sections as a grey-level volume images. For models that have delineated anatomy, the model includes a set of domain images, one for each delineated anatomical component	Images in woolz format. Other formats using Woolz tools
High-resolution section images	Each model is created by 3D reconstruction from serial histological sections. The original 2D images at high resolution are available for download	Zip file with jpeg format images
eHistology image	Large high-resolution 2D images of the requested section plus data files providing the embryo detail, annotation list, EMAPA IDs with coordinate locations for each term	Zip file containing tiff image plus text files for metadata
Raw expression data	Each assay provides a link to the raw data used to create the entry. 3D data are primarily OPT	3D data in woolz format. 2D data in jpeg, tiff, or png
Expression domain	As data are mapped a binary representation of the expression, pattern in the target model is created. Images of the mapped pattern can be downloaded from the website	Png format 3D image
Movies	Many movies have been generated for the atlas models and the 3D-mapped gene expression data. These are available for direct play in the browser via YouTube or for download	Download format mpeg (mp4)

## Discussion

EMAP is now a long-term project, and the full resource has been available since 2001. Since then, there has been a dramatic increase in the number of gene expression entries and the range of the atlas models from a few hundred patterns and eight delineated models, to 28 K plus entries, a full range of models at all post-implantation Theiler stages, and an ontology that is the de facto standard for mouse embryo data which has become an independent community resource. The current interfaces allow exploration of the data with visualisation and query in the context of the atlas model framework. The recently released eHistology resource extends the functionality to high-resolution cellular level histology coupled with the annotations from the definitive Atlas of Mouse Development by Kaufman. The underlying technical developments are also becoming more widely used as the demand for online visualisation of large-scale 3D images and image archives grows, and become embedded in atlas-based systems for research and teaching.
